# Deep learning–empowered low‐cost portable automated refraction system: A solution to the inadequate effective correction rate of refractive errors in resource‐limited areas

**DOI:** 10.1111/opo.70027

**Published:** 2025-10-24

**Authors:** Huang Yan, Zhen Yi, Sun Qilin, Chang Hong, Huang Yan, Tang Wei

**Affiliations:** ^1^ National Engineering Research Centre for Ophthalmology, Beijing Institute of Ophthalmology, Beijing Tongren Eye Centre, Engineering Research Centre of the Ministry of Education for Ophthalmic Diagnosis and Treatment Equipment and Materials, Beijing Key Laboratory of Ophthalmology and Visual Science, Beijing Tongren Hospital Capital Medical University Beijing China; ^2^ State Key Laboratory of Multimodal Artificial Intelligence Systems Institute of Automation, Chinese Academy of Sciences (CASIA) Beijing China; ^3^ Beijing, He Eye Specialist Hospital Beijing China

**Keywords:** deep learning, effective correction rate, low‐cost, refractive errors, resource‐limited areas

## Abstract

**Aims:**

To develop and validate a low‐cost, portable automated refraction system (Tongren Digital Sight, TRDS) using deep learning and infrared eccentric photorefraction to improve refractive error correction in resource‐constrained settings.

**Methods:**

This randomised controlled crossover trial enrolled 282 participants (18–60 years of age) at Beijing Tongren Hospital. TRDS utilised low‐cost components (complementary metal‐oxide semiconductor (CMOS) detector, infrared light emitting diodes) and an end‐to‐end deep learning model trained on 362,000 images to analyse fundus reflection signals. Primary outcomes included agreement between TRDS and subjective refraction (gold standard) via Pearson correlation, Bland–Altman plots and intraclass correlation coefficients (ICC) for spherical equivalent (*M*), horizontal (*J*_0_) and oblique (*J*_45_) cylinder parameters. Secondary outcomes included corrected visual acuity.

**Results:**

TRDS showed strong correlations with subjective refraction: *M* (*r* = 0.96, ICC = 0.96), *J*_0_ (*r* = 0.74, ICC = 0.71) and *J*_45_ (*r* = 0.76, ICC = 0.75). Mean differences were 0.07 D (*M*), −0.06 D (*J*_0_) and 0.00 D (*J*_45_), with 95% limits of agreement within clinical tolerance. Corrected visual acuity was non‐inferior to that of subjective refraction (mean difference = 0.03 logMAR, *p* < 0.01). Subgroup analysis showed consistent performance across myopia and hyperopia, except for moderate agreement in high myopia (*M*: *r* = 0.62). Subjective and TRDS refraction showed corrected visual acuity of 0.01 (SD = 0.13) and −0.02 (SD = 0.08) logMAR, respectively, with a significant mean difference of 0.03 logMAR (95% LoA: 0.01–0.04, *p* < 0.01). Corrected visual acuity >0.18 logMAR occurred in 13 (4.6%) and 14 (5.0%) participants for subjective and TRDS refraction, respectively.

**Conclusions:**

The deep learning‐empowered TRDS system demonstrated high accuracy and portability. It offers a scalable solution to improve refractive error correction rates, aligning with WHO goals to enhance global eye health.

**Key points**

- This study introduces a deep learning‐based refraction system that provides accurate prescriptions using low‐cost hardware.
- By enabling task‐shifting and reduced reliance on expensive optics, this approach expands accessible eye care and advances progress towards vision health targets.
- The device demonstrated strong agreement with standard clinical refraction and reliable visual outcomes, supporting its potential for large‐scale clinical application.

## INTRODUCTION

Refractive error, being one of the major causes of visual impairment and blindness globally, significantly affects people's quality of life and socioeconomic development.^1^, ^2^ In low‐ and middle‐income regions, the scarcity of medical resources and professionals has led to a persistently low correction rate for refractive errors, imposing a burden on both patients and society.^3^, ^4^ Based on data from the World Health Organization (WHO), approximately 1.84 billion people suffer from moderate to severe visual impairment or blindness due to myopia, and the global median effective refractive error correction coverage (eREC) is only 35.7%.^5^ The 73rd World Health Assembly passed a resolution requiring an increase of 40 percentage points in eREC by 2030.^6^, ^7^

Subjective refraction, the gold standard for obtaining refractive error correction prescriptions, is excellent in terms of accuracy and reliability. However, it has issues such as being time‐consuming and highly dependent on optometrists. In developing countries, the shortage of optometrists has become the main bottleneck in improving eREC.^8^ To address these problems, in recent years, technological innovations have mainly revolved around remote refraction systems and automated spectacle‐fitting systems.

Remote refraction has proven to be an effective way to increase eREC. However, current remote refraction models have not achieved complete independence from optometrists. There are problems such as a disconnection between the screening and spectacle‐fitting processes, a high misdiagnosis rate in community screenings and insufficient accessibility in remote areas.^9^ To ensure the accuracy of the measurements, automated refraction devices usually rely on expensive photosensitive components and a precise and stable focusing system, posing a severe challenge to their popularisation in low‐income regions.^10^ Although portable automated refractors such as the Nikon Retinomax (nikon.com) are commercially available, they are relatively expensive.^11^, ^12^ A growing body of evidence shows that several low‐cost or portable autorefractors can achieve clinically acceptable agreement with subjective refraction (SR) under defined conditions, often with good patient acceptance in randomised crossover designs.^13^, ^14^, ^15^, ^16^ At the same time, performance varies across devices, refractive subgroups and testing environments. Additionally, several reports have indicated that the introduction of open‐field, non‐cycloplegic autorefraction remains challenging.^17^, ^18^, ^19^, ^20^

The emergence of end‐to‐end deep learning models offers an opportunity to solve these problems. Rather than asserting uniform inferiority of existing solutions, the authors frame the present work as addressing a specific gap: Whether an end‐to‐end deep‐learning pipeline can maintain SR‐level agreement using ultra‐low‐cost hardware and in open‐field conditions, thereby enabling task shifting with minimal operator training. This type of model has a powerful ability to extract image features automatically, which can greatly reduce the requirements for hardware performance.

This study combines this technology with infrared eccentric photorefraction, uses low‐cost components and optimises the imaging algorithm. The aim was to develop a device for generating refractive error correction prescriptions cost‐effectively, with high accuracy and reliability in resource‐constrained areas, verify whether its accuracy meets clinical standards, promote the realisation of global eye health goals and respond to the eye health plan proposed by the World Health Organization in 2021.

## MATERIALS AND METHODS

This study adopted a diagnostic test research method. The research protocol was approved by the Ethics Review Committee of Beijing Tongren Hospital, Capital Medical University. Before implementation of the study, written informed consent was obtained from all research participants and the principles of the Declaration of Helsinki were followed. The research protocol has been registered on ClinicalTrials.org (Registration number: ChiCTR2500100874).

### Design and participants

This was a randomised controlled crossover clinical trial conducted at Beijing Tongren Hospital, Capital Medical University, from March 2025 to April 2025. Volunteers were between 18 and 60 years of age and came from the general ophthalmology outpatient department of Beijing Tongren Hospital due to refractive errors. Research participants were limited to 18–60 years of age because individuals under 18 years old have strong accommodation, which increases the complexity of spectacle fitting. The following exclusion criteria were applied: (1) refractive error beyond the detection range of the device (−10 to +10 dioptres [D]); (2) cylindrical lens power > 3D (patients are often reluctant to wear spectacles when high astigmatism is fully corrected); and (3) having speech or hearing impairments.

### Development of the deep learning‐empowered low‐cost portable automated refraction system

From an algorithmic perspective, existing commercial portable autorefractors measure refractive error using traditional image‐processing methods. The process sequentially applies conventional techniques to fit and locate the pupil, calculate either the pupil's brightness gradient or the area of the crescent‐shaped reflex and then computes refractive error via a linear mapping model built from those features. This multi‐stage, multi‐module pipeline can lead to cumulative errors. Further, the linear mapping model is extremely sensitive to input variations, and minor changes can produce vastly different outputs. Therefore, achieving accurate refractive measurements demands very stringent environmental conditions, such as controlled lighting and minimal occlusion of the eye being tested. Finally, these traditional methods impose harsh requirements on imaging conditions and often fail in real‐world use. For example, when ambient light is bright and the pupil constricts, current commercial portable autorefractors may be unable to deliver measurements.

**FIGURE 1:**
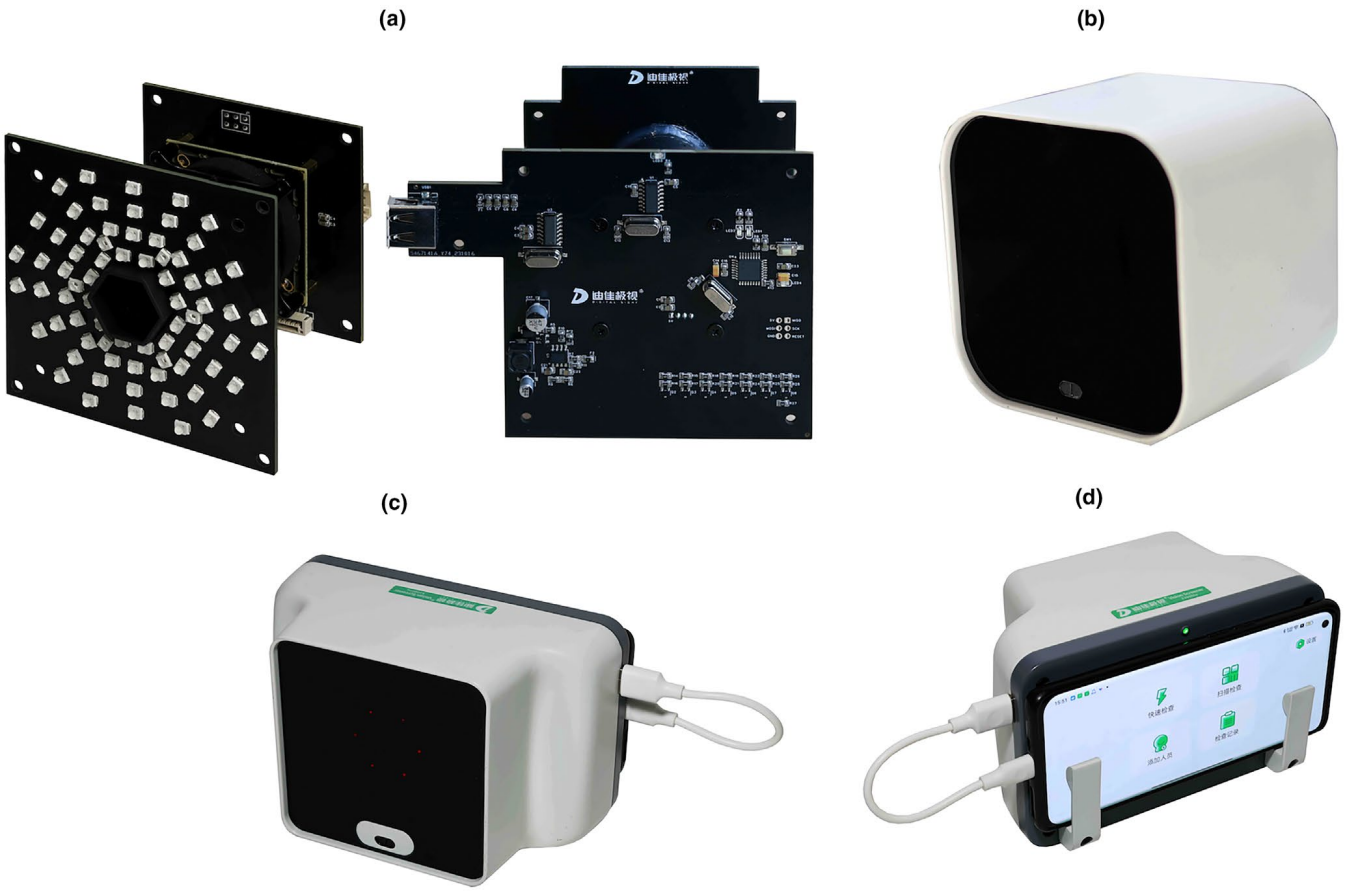
Hardware design of the TRDS Prototype (Subplot a illustrates the internal structure of TRDS, Subplot b displays the TRDS connected to a computer and smart TV and Subplots c and d showcase the TRDS connected to a mobile phone). TRDS, Tongren Digital Sight.

In this study, an innovative and highly simplified infrared eccentric refractometer (Tongren Digital Sight, TRDS) was developed. Differing from traditional infrared eccentric refractometers, the TRDS uses low‐cost components, a simplified optical layout and completely removes moving parts. Its open‐field‐of‐view design reduces the instrument‐induced myopia (see Figure 1). The TRDS prototype has several innovative designs and differs significantly from traditional infrared eccentric refractometers. Firstly, the TRDS uses infrared light‐emitting diodes (LEDs) as the light source rather than expensive super‐luminescent diodes, thereby reducing the cost of the light source components significantly. Secondly, a low‐cost Complementary Metal–Oxide–Semiconductor (CMOS) detector was selected to replace the scientific‐grade charge‐coupled device as the wave‐front sensor, further reducing the hardware cost. Finally, the TRDS was connected to a mobile phone through a dedicated Printed Circuit Board (PCB) circuit and an Internet‐of‐Things module; the power and chip of the mobile phone were used to upload and download data. This hardware design greatly simplifies the overall layout of the TRDS, significantly reduces the material and manufacturing costs (less than USD150) and improves the portability and cost‐performance of the device. The TRDS connects to a smartphone via a USB‐C port. The accompanying software is currently compatible with Android phones, and support for Apple (iOS) devices will be added in the future.

**FIGURE 2:**
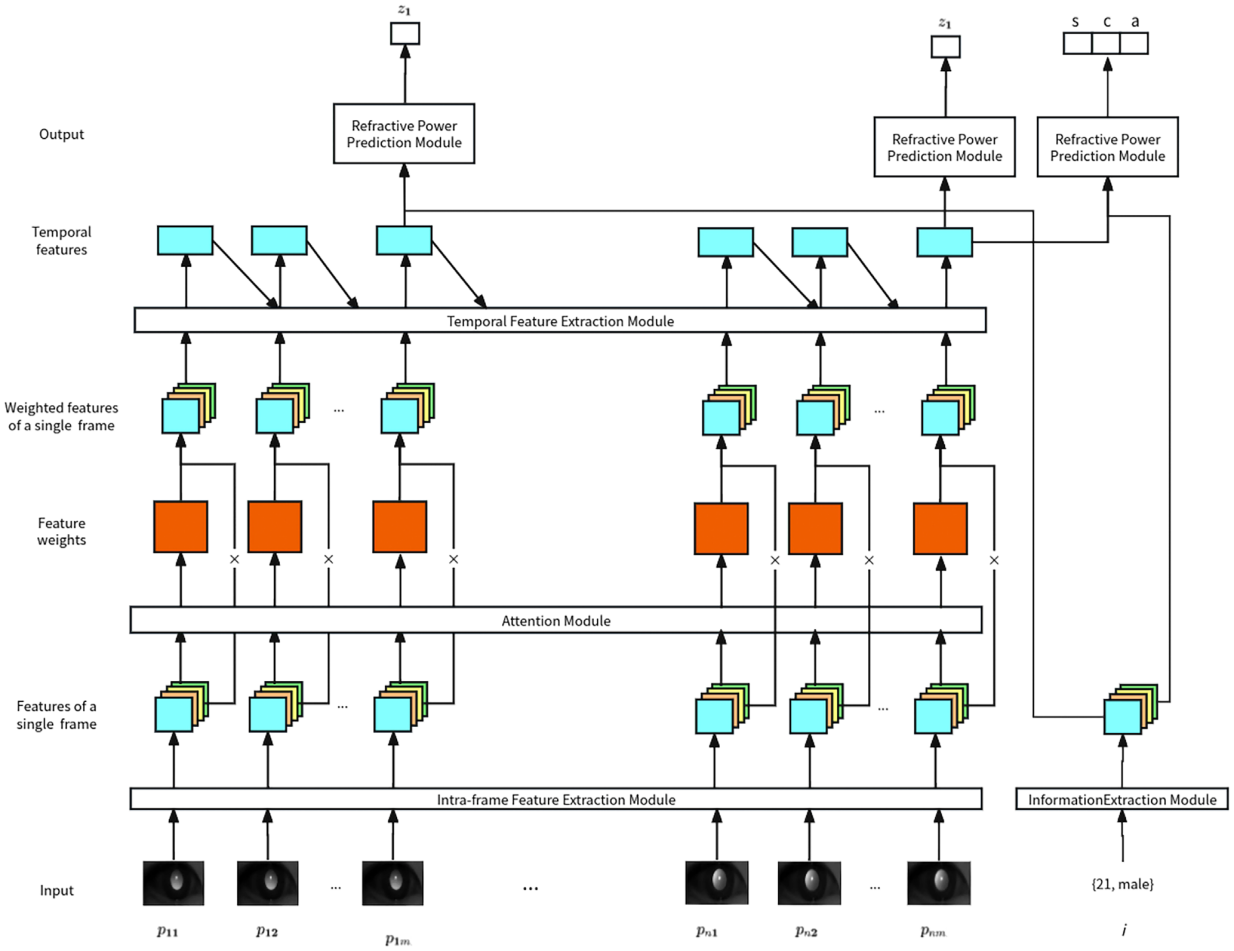
Development of the refraction calculation algorithm based on deep learning. Z1 is the refractive power along a single meridian, predicted by the model from the corresponding infrared eccentric photorefraction image. S, C, and A—the sphere, cylinder, and axis—are predicted by the model from a series of meridian‐wise infrared eccentric photorefraction images.

To make up for the possible performance limitations of low‐cost components, this study adopted active sensing technology and deep‐learning algorithms to analyse fundus reflection images and calculate the magnitude of refractive error (Figure 2). Firstly, to achieve accurate detection of ametropia and overcome the limitations of the dataset, a high‐quality infrared eccentric photorefraction image dataset was constructed and annotated. Image sequences (input X) were collected from volunteers using the TRDS and true Y values were obtained through standard subjective refraction, thus forming a dataset *D* = (*X,Y*). During the data collection process, volunteers wore lenses with different optical powers to simulate varying refractive states, increasing the diversity of the output.

To ensure the robustness of the model in various refractive scenarios, a diverse dataset was carefully designed covering a wide range of conditions. The ambient light was adjusted to collect images with different pupil diameters. The dataset also included ocular images with different characteristics, such as variations in eyelash length, ptosis and degree of occlusion, thereby enhancing the model's adaptability to external factors. In addition, the participants of different ages and genders were tested, ensuring the model's universality among different populations. A highlight of this part of the work is the unprecedented scale of the dataset, which contained 362,000 infrared eccentric photorefraction images taken under different conditions. We believe this is the largest dataset of its kind. Prior to the start of this clinical trial, the aforementioned image acquisition was completed for model training. Healthy volunteers were recruited, and each participant sequentially wore seven sets of trial lenses of different powers to simulate multi‐level refractive errors, while pupil constriction and dilation were induced under four ambient lighting conditions. Imaging was performed using a device equipped with six directions of eccentric illumination, capturing four frames per direction. After quality screening to remove frames with closed or blinking eyes, 362,000 high‐quality infrared eccentric images were obtained, providing a multidimensional dataset covering seven refractive levels and four pupil conditions for subsequent deep‐learning model training.

The dataset was divided into a training and a validation set in a 4:1 ratio. All images were resized to 256 × 256 pixels before being input into the model. To increase data diversity further and reduce overfitting, data augmentation techniques were adopted, such as random rotation, vertical and horizontal flipping. This large and diverse dataset plays a crucial role in ensuring the robustness and accuracy of the proposed model.

The training and testing of the model were carried out on a system equipped with the Ubuntu 22.04 (Canonical Ltd., ubuntu.com/), Python 3.8.19 (Python Software Foundation, python.org/), CUDA 11.8 (NVIDIA Corporation, developer.nvidia.com/cuda‐toolkit) and a NVIDIA GeForce RTX 4090 GPU (NVIDIA Corporation, nvidia.com). The Stochastic Gradient Descent (SGD) algorithm^21^ was used, with a momentum set to 0.95, an initial learning rate of 1e^2^ and a Step Decay learning rate scheduler with a 0.9 weight decay. The training batch size was eight, the number of training epochs was 300 and the loss function was the Mean Squared Error (MSE). Finally, the model with the minimum validation set loss was selected as the final model for dioptric prediction.

Compared with existing commercial portable autorefractors, this study proposes an end‐to‐end deep learning method for refractive error calculation. A set of ocular images captured by an infrared eccentric camera is fed into a convolutional neural network that directly predicts the subject's refractive error. We also meticulously collected a multi‐scenario infrared eccentric photorefraction dataset. The Convolutional Neural Network (CNN) model is then trained on this large‐scale dataset to learn both image features automatically and the mapping from those features to refractive values. The specific advantages are:

- Improved accuracy: The deep CNN, trained on large‐scale data, can automatically learn image features and the feature‐to‐refraction mapping, fitting real‐world data more accurately than traditional image‐processing methods.
- Simplified pipeline: The end‐to‐end model has fewer stages, avoiding cumulative errors arising from multiple processing modules.
- Greater robustness: As a highly non‐linear model, the deep learning approach is more resilient to variations in illumination, pupil diameter and other ocular changes than the linear models used in current commercial portable autorefractors, reducing the demands on imaging component performance.

To ensure measurement accuracy, the proposed end‐to‐end method imposes certain performance requirements on the hardware. Therefore, a hybrid local + cloud computing architecture was adopted; the device performs local preprocessing and uploads the data to the cloud, where the end‐to‐end refractive calculation is executed. This shifts 90% of the computational load to the cloud, allowing lower specification local hardware without compromising the user experience. A single cloud server can support over 10,000 devices, making this architecture more cost‐effective than traditional devices, each of which must bear the full computational cost. Moreover, through model compression techniques, a compact version of the model can be deployed on low‐cost mobile devices for preliminary refractive screening, enabling offline operation without network or cloud dependence.

### Sample size

In this study, a crossover self‐control design was adopted, in which each subject randomly received two different treatments: TRDS automated refraction and standard subjective refraction. The sample size was calculated by substituting the results of the pre‐experiment (the mean of the equivalent spherical lens difference between the two refraction methods was 0.07 and the standard deviation was 0.24), the significance level (set to 0.05) and the test power (set to 0.80) into the following formula:

$$n={\left(\frac{Z_{1-a/2}+Z_{1-\beta }}{\delta /\sigma }\right)}^{2}$$

where n is the number of participants; $Z_{1-\alpha /2}$ is the standard normal quantile for the two‐sided significance level α; $Z_{1-\beta }$ is the standard normal quantile for power 1−β; δ is the mean paired difference to be detected (target effect size) and σ is the standard deviation of the paired differences from the pilot study.

$$n={\left(\frac{1.96+0.84}{0.07/0.24}\right)}^{2}\approx 92$$

Rounding up to the nearest integer, at least 92 samples were required. Assuming a dropout rate of 20%, at least 110 samples were needed.

### Research procedures

This study was conducted in the ophthalmology outpatient department of Beijing Tongren Hospital, Capital Medical University, following the standard refraction process. Participants underwent refractive assessments using three methods—TRDS, autorefractor (AR, fully automatic autorefractor/keratometer (ARK‐F), NIDEK and nidek‐intl.com) followed by subjective refraction and VS100 Spot Vision Screener (VS100, Welch Allyn, hillrom.com/en/products/spot‐vision‐screener)—in a randomised order. The VS100 was chosen because it uses the same refraction measurement principle as the TRDS. The randomisation sequence for these three methods was generated by a research statistician. Using the ‘random permutation’ function in IBM SPSS software (ibm.com), the statistician created a unique random order of the three methods for each participant, ensuring that all six possible permutations were assigned to participants in appropriate proportions to balance potential order‐related biases. For instance, some participants received the sequence ‘TRDS → autorefractor followed by subjective refraction → VS100’, while others followed ‘VS100 → TRDS → autorefractor followed by subjective refraction’, among other permutations.

The generated random sequences were stored separately by the statistician and sealed in opaque envelopes. Only when a participant entered the refraction process did the research assistant, who was not involved in refraction operations or the evaluation of the results, unseal the envelopes in sequence to obtain the assigned order. Technicians performing the refractions were only informed of the current refraction method to be conducted, with no knowledge of the subsequent order or the arrangements for other participants, thus avoiding potential biases caused by subjective expectations.

The subjective refraction was carried out based on a standard process. All tests were completed in a dark room using a phoropter combined with a standard logarithmic visual acuity chart; the test distance was set at 5 m. The testing process was divided into two stages: monocular correction and binocular balance. First, the initial spherical power from the autorefractor was refined using the red–green bichrome test to balance clarity between the two colours, thereby determining the spherical endpoint with the minimum minus: best‐corrected visual acuity (BCVA). This was confirmed on a standard logMAR chart with that endpoint. Subsequently, the cross cylinder method was performed using a ±0.50D Jackson Cross Cylinder, and the astigmatic axis and magnitude were determined after three endpoint judgements. In the binocular balance stage, the prism dissociation technique was applied, and the spherical lens power was adjusted to make the clarity of the two eyes approach equality. Finally, the stability of the prescription was verified by the fogging method. After adding +0.75 D of fog, the lens power was gradually reduced back to the stable BCVA value. All objective and subjective refractions were performed by the same optometrist.

### Provision of trial spectacle and follow‐up

The subjective refraction and TRDS automated refraction findings were input into the research database. Different random number codes were used to distinguish the two prescriptions for analysis. The random number codes were generated by a research statistician from the clinical trial department of Beijing Tongren Hospital for each participant through a computer. An independent and blinded optometrist prepared trial spectacles with two pairs of trial frames and lenses having the same appearance, based on the subjective and TRDS objective refraction findings. Volunteers, the assemblers of the trial spectacles and the final evaluators, were all unaware of the source of the prescriptions. Volunteers were randomly assigned to wear a pair of trial spectacles for 15 min and then underwent a visual acuity test. Volunteers returned the first pair of spectacles, rested for 3 h, received a second pair of spectacles, wore them for 15 min and then returned for the second follow‐up evaluation.

### Outcome measures

The primary outcome measure was the level of agreement between the TRDS automated refraction and subjective refraction prescriptions. The secondary outcome measure was the difference in corrected visual acuity based on the two refractive findings.

### Statistical analysis

For statistical comparison, the refractive prescriptions were converted into the power vectors of spherical equivalent (*M*), horizontal Jackson Cross Cylinder (*J*_0_) and oblique Jackson Cross Cylinder (*J*_45_), from the subjective refraction (M_SR_, *J*_0,SR_ and *J*_45,SR_), TRDS (M_AR_, *J*_0,AR_ and *J*_45,AR_) and VS100 eccentric refraction (M_VS_, *J*_0,VS_ and *J*_45,VS_) findings. Pearson correlation coefficient (*r*), Bland–Altman plots and the intraclass correlation coefficient (ICC) were used to analyse the correlation and agreement between the measurement results, including overall analysis and sub‐analysis by refractive error groups. The refractive error subgroups were defined as low myopia (>−3 D), moderate myopia (−3 to −6 D), high myopia (<−6D), low hyperopia (hyperopia <+1 D) and high hyperopia (hyperopia ≥ +1 D). Visual acuity was measured using a Snellen chart at a test distance of 5 m, and all measurements were converted into logMAR (logarithm of the minimum angle of resolution) units for statistical comparison. The refraction results of the right and left eyes were analysed separately. However, only the right eye findings are reported because the results of the two eyes were similar. The significance level was set at *p* ≤ 0.05, and all statistical analyses were performed using IBM SPSS 23.0 (ibm.com/products/spss).

## RESULTS

### Participants

A total of 282 participants underwent refractive examinations based on automated and subjective refraction and received corresponding spectacle prescriptions. The mean (SD) age was 28.8 (3.0) years and 57.1% were female. Mean (SD) uncorrected visual acuity was 0.44 (0.29) logMAR. The results of the participants in terms of demographic, refractive error and visual acuity characteristics are shown in Table 1.

**TABLE 1:** Demographic and vision characteristics of participants.

Patient characteristics	*N* = 282
Age in years	28.8 (3.0)
Female, *N* (%)	161 (57.1%)
Right eye presenting visual acuity (logMAR)	0.44 (0.29)
*M*_SR_	−2.83 (3.04)
*J*_0, SR_	0.26 (0.47)
*J*_45, SR_	0.02 (0.24)
*M*_AR_	−2.91 (3.03)
*J*_0, AR_	0.32 (0.37)
*J*_45, AR_	0.02 (0.20)
*M*_VS_	−2.38 (2.51)
*J*_0, VS_	0.15 (0.31)
*J*_45, VS_	0.00 (0.20)

*Note*: Results are shown as mean (SD).

Abbreviations: AR, autorefraction; BCVA, best‐corrected visual acuity; *J*_0_, horizontal Jackson Cross Cylinder; *J*_45_, oblique Jackson Cross Cylinder; logMAR, logarithm of the minimum angle of resolution; *M*, spherical equivalent; *M*, *J*_0_ and *J*_45_ values are shown in dioptres; SR, subjective refraction; VS, VS100 Spot Vision Screener.

### Agreement between subjective refraction, TRDS automated refraction and corrected visual acuity

**FIGURE 3:**
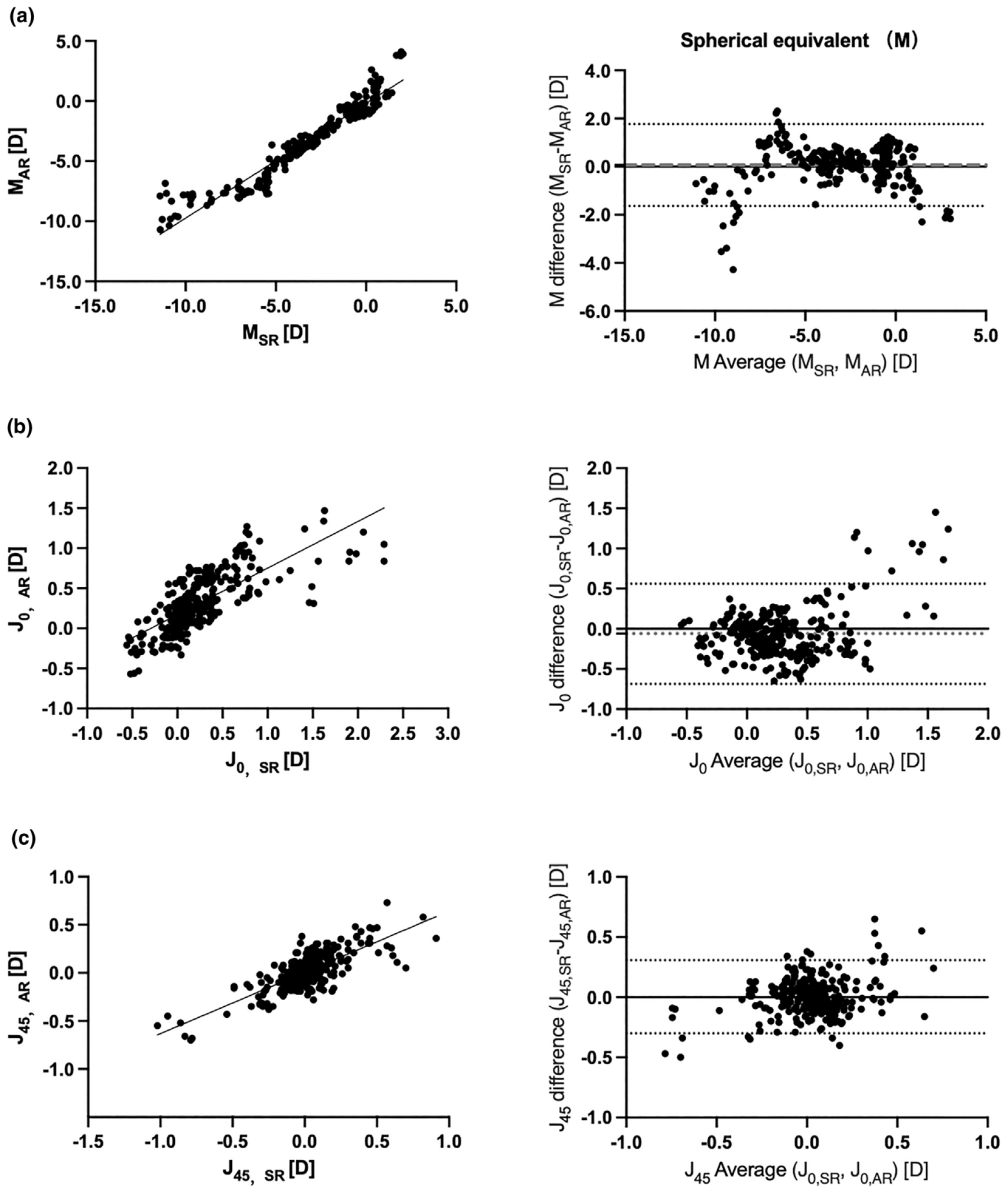
Correlation (left) and Bland–Altman (right) plots comparing agreement of prescriptions measured by SR and AR. Panels show (a) spherical equivalent, *M*, (b) horizontal Jackson Cross Cylinder, *J*_0_ and (c) oblique Jackson Cross Cylinder, *J*_45_. AR, artificial intelligence autorefraction; D, dioptres; SR, subjective refraction.

There was a strong correlation between subjective and TRDS automated refraction. The Pearson correlation coefficients for *M*, *J*_0_ and *J*_45_ were *r* = 0.96 (*p* < 0.01), *r* = 0.74 (*p* < 0.01) and *r* = 0.76 (*p* < 0.01), respectively. The mean difference in dioptres was 0.07 (95% limits of agreement (LoA), −1.64 to 1.77), −0.06 (95% LoA, −0.69 to 0.56), and 0.00 (95% LoA, −0.30 to 0.31), respectively (Table 2 and Figure 3). The intraclass correlation coefficients (ICC) also showed a high degree of agreement. The ICCs for *M*, *J*_0_ and *J*_45_ were 0.96 (95% confidence interval [CI], 0.95–0.97), 0.71 (95% CI, 0.65–0.76) and 0.75 (95% CI, 0.70–0.80), respectively. In the subgroup analysis by refractive error subcategories, there was a strong correlation between subjective refraction and TRDS automated refraction prescriptions, with correlation coefficients *r* ranging from 0.70 (*p* < 0.01) to 0.96 (*p* < 0.01), except for the values of *M* in the high myopia group, *J*_45_ in the moderate myopia group, as well as *M* and *J*_45_ in the low hyperopia group.

**TABLE 2:** Comparison of subjective refraction and Tongren Digital Sight (TRDS) autorefraction (*N* = 282).

Refractive error	Refractive parameter	Subjective mean (SD)	Autorefraction mean (SD)	Mean difference	95% LOA	*r*	ICC
All, *N* = 282	*M*	−2.84 (3.04)	−2.91 (3.03)	0.07	−1.64 ~ 1.77	0.96	0.96
	*J*_0_	0.26 (0.47)	0.32 (0.37)	−0.06	−0.69 ~ 0.56	0.74	0.71
	*J*_45_	0.02 (0.24)	0.02 (0.20)	0.00	−0.30 ~ 0.31	0.76	0.75
High myopia ≤ −6 D, *N* = 39	*M*	−8.59 (1.80)	−8.07 (0.94)	−0.52	−3.30 ~ 2.26	0.62	0.51
	*J*_*0*_	0.86 (0.85)	0.50 (0.44)	0.36	−0.65 ~ 1.36	0.87	0.71
	*J*_45_	0.08 (0.48)	0.05 (0.27)	0.03	−0.53 ~ 0.58	0.87	0.74
Moderate myopia −3 to −6 D, *N* = 76	*M*	−4.41 (0.84)	−4.78 (1.28)	0.38	−0.93 ~ 1.68	0.88	0.81
	*J*_*0*_	0.26 (0.28)	0.52 (0.28)	−0.26	−0.60 ~ 0.09	0.80	0.80
	*J*_45_	0.05 (0.13)	0.03 (0.17)	0.01	−0.28 ~ 0.31	0.53	0.51
Low myopia >−3 D, *N* = 125	*M*	−1.29 (0.92)	−1.44 (1.02)	0.15	−0.72 ~ 1.03	0.90	0.89
	*J*_*0*_	0.16 (0.27)	0.22 (0.33)	−0.06	−0.43 ~ 0.30	0.83	0.81
	*J*_45_	0.02 (0.16)	0.02 (0.17)	0.00	−0.18 ~ 0.17	0.85	0.85
Low hyperopia <+1 D, *N* = 32	*M*	0.43 (0.20)	0.41 (1.04)	0.02	−1.86 ~ 1.90	0.48	0.18
	*J*_*0*_	−0.04 (0.20)	0.11 (0.28)	−0.15	−0.54 ~ 0.24	0.70	0.67
	*J*_45_	0.01 (0.16)	0.02 (0.22)	−0.01	−0.33 ~ 0.31	0.67	0.63
High hyperopia ≥+1 D, *N* = 10	*M*	1.62 (0.37)	2.55 (1.73)	−0.93	−3.63 ~ 1.78	0.95	0.39
	*J*_*0*_	0.00 (0.20)	−0.07 (0.33)	0.07	−0.38 ~ 0.51	0.74	0.65
	*J*_45_	−0.22 (0.35)	−0.16 (0.32)	−0.06	−0.26 ~ 0.14	0.96	0.95

Abbreviations: D, dioptres; ICC, intraclass correlation coefficients; *J*_45_, oblique Jackson Cross Cylinder; *J*_0_, horizontal Jackson Cross Cylinder; LOA, limits of agreement; *M*, spherical equivalent; *r*, Pearson correlation coefficient; SD, standard deviation.

Corrected visual acuities with the subjective and TRDS automated refraction prescriptions were 0.01 (0.13) and −0.02 (0.08), respectively. The difference between the two was 0.03 (95% LoA 0.01–0.04, *p* < 0.01). The corrected visual acuities of 13 participants (4.6%) with the subjective refraction prescription and 14 participants (5.0%) with the TRDS automated refraction were worse than 0.18 logMAR.

### Agreement between subjective refraction and the VS100 infrared eccentric objective refraction

**FIGURE 4:**
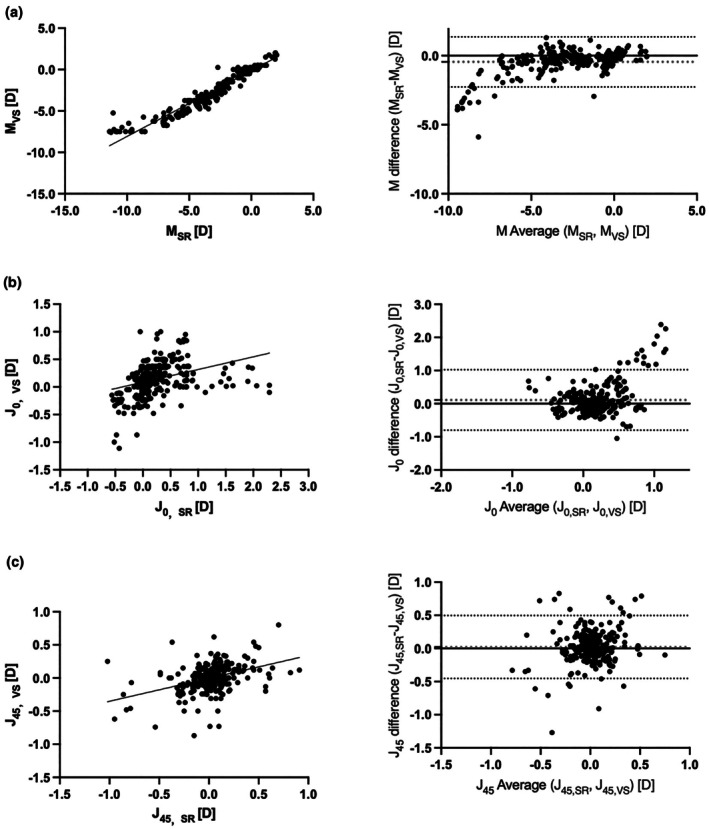
Correlation (left) and Bland–Altman (right) plots comparing agreement of prescriptions measured by SR and VS. Panels show (a) spherical equivalent, *M*, (b) horizontal Jackson Cross Cylinder, *J*_0_ and (c) oblique Jackson Cross Cylinder, *J*_45_. D, dioptres; SR, subjective refraction; VS, VS100 Spot Vision Screener.

A strong correlation was only observed between the *M* value for the subjective and VS100 infrared eccentric objective refraction and only a weak correlation between *J*_0_ and *J*_45_. Pearson correlation coefficients were 0.96 (*p* < 0.01), 0.34 (*p* < 0.01) and 0.40 (*p* < 0.01), respectively. The mean differences in dioptres were −0.45 (95% LoA, −2.27 to −1.36), 0.11 (95% LoA, −0.80 to 1.02) and 0.02 (95% LoA, −0.45 to 0.50), respectively (Table 3 and Figure 4). The intraclass correlation coefficients (ICC) for *M*, *J*_0_ and *J*_45_ were 0.95 (95% CI, 0.93–0.96), 0.32 (95% CI, 0.21–0.42) and 0.40 (95% CI, 0.30–0.49), respectively. In the subgroup analysis by refractive error categories, only *M* in the moderate myopia group (*r* = 0.81, *p* < 0.01), *M* in the low myopia group (*r* = 0.88, *p* < 0.01), *J*_0_ in the low myopia group (*r* = 0.78, *p* < 0.01) and *J*_0_ in the high hyperopia group (*r* = 0.76, *p* < 0.01) showed strong correlations, and the remainder were moderately or weakly correlated. The agreement with the subjective refraction prescription was lower than that of the TRDS automated refraction prescription.

**TABLE 3:** Comparison of subjective refraction and Welch Allyn VS100 spot vision screener (*N* = 282).

Type of refractive error	Refractive parameter	Subjective mean (SD)	Vision screener mean (SD)	Mean difference	95% LOA	*r*	ICC
All, *N* = 282	*M*	−2.84 (3.04)	−2.38 (2.51)	−0.45	−2.27 ~ 1.36	0.96	0.95
	*J*_*0*_	0.26 (0.47)	0.15 (0.31)	0.11	−0.80 ~ 1.02	0.34	0.32
	*J*_45_	0.02 (0.24)	0.00 (0.20)	0.02	−0.45 ~ 0.50	0.40	0.40
High myopia ≤ −6 D, *N* = 39	*M*	−8.59 (1.80)	−6.62 (0.86)	−1.97	−4.66 ~ 0.73	0.67	0.52
	*J*_*0*_	0.86 (0.85)	0.03 (0.21)	0.83	−0.59 ~ 2.26	0.66	0.31
	*J*_45_	0.08 (0.48)	0.08 (0.48)	0.07	−0.78 ~ 0.92	0.45	0.36
Moderate myopia −3 to −6 D, *N* = 76	*M*	−4.41 (0.84)	−4.11 (0.83)	−0.3	−1.32 ~ 0.72	0.81	0.81
	*J*_*0*_	0.26 (0.28)	0.10 (0.27)	0.16	−0.35 ~ 0.68	0.54	0.54
	*J*_45_	0.05 (0.13)	0.00 (0.21)	0.05	−0.35 ~ 0.45	0.37	0.33
Low myopia > −3 D, *N* = 125	*M*	−1.29 (0.92)	−0.99 (1.12)	−0.29	−1.33 ~ 0.75	0.88	0.87
	*J*_*0*_	0.16 (0.27)	0.20 (0.34)	−0.04	−0.45 ~ 0.38	0.78	0.76
	*J*_45_	0.02 (0.16)	0.01 (0.19)	0.01	−0.36 ~ 0.38	0.41	0.41
Low hyperopia <+1 D, *N* = 32	*M*	0.43 (0.20)	0.25 (0.17)	0.18	−0.18 ~ 0.55	0.49	0.48
	*J*_*0*_	−0.04 (0.20)	0.17 (0.30)	−0.21	−0.67 ~ 0.24	0.62	0.58
	*J*_45_	0.01 (0.16)	0.04 (0.11)	−0.03	−0.35 ~ 0.30	0.29	0.27
High hyperopia ≥ +1 D, *N* = 10	*M*	1.62 (0.37)	1.43 (0.54)	0.2	−0.57 ~ 0.96	0.69	0.65
	*J*_*0*_	0.00 (0.20)	0.17 (0.44)	−0.17	−0.79 ~ 0.46	0.76	0.57
	*J*_45_	−0.22 (0.35)	−0.21 (0.29)	−0.01	−0.57 ~ 0.55	0.62	0.61

Abbreviations: D, dioptres; ICC, intraclass correlation coefficients; *J*_45_, oblique Jackson Cross Cylinder; *J*_0_, horizontal Jackson Cross Cylinder; LOA, limits of agreement; *M*, spherical equivalent; *r*, Pearson correlation coefficient; SD, standard deviation.

## DISCUSSION

This study developed a deep learning‐empowered, low‐cost, portable automated refraction system (TRDS), providing an innovative solution to the problem of low refractive error correction rates where optometrists are scarce. The results show that the TRDS findings exhibit a strong correlation with subjective refraction in terms of *M*, *J*_0_ and *J*_45_ (Pearson correlation coefficients were 0.96, 0.74 and 0.76, respectively), and the corrected visual acuity was equivalent to that of subjective refraction, thus confirming its feasibility and accuracy in clinical applications.

The scarcity of optometrists is a bottleneck in improving eREC.^22^ In recent years, technological innovations have addressed this problem through two approaches: remote and automated refraction. Health economic studies have confirmed that integrating remote refraction into the primary healthcare system is an effective way to improve eREC.^23^, ^24^, ^25^ A high degree of agreement has been shown between remote and in‐person refraction. For the spherical powers, 84.6% fell within 0.5D of the value determined by an optometrist, while for the cylinder axis, 74% lay within the range deemed acceptable, that is, within 10°. Further, best‐corrected visual acuity matched in 82% of the eyes, and the difference was within 0.10 logMAR in 92% of the eyes.^26^ A validation study of the Peek Solutions (Peek Vision Ltd., peekvision.org) software in Kenya showed that it can effectively improve eREC, but remote refraction still depends on optometrists, resulting in a broken service chain and limited coverage efficiency.^27^, ^28^, ^29^

Otero et al.^30^ transformed traditional manual phoropters into automated instruments through innovative designs using motors and customised software. The Essilor Vision‐R800 (Essilor Instruments, essilor‐instruments.com) refractometer can evaluate spherical and cylindrical lens powers simultaneously through adjustable lenses and advanced algorithms, significantly shortening the refraction time.^31^ There are other automated refraction systems such as Nidek TS‐610 (NIDEK Co., Ltd., nidek‐intl.com), Vision Fit (Adaptica S.r.l., adaptica.com) and Eye Refract (Visionix, visionix.com), but the high cost of these devices makes it difficult to popularise them in resource‐limited areas.^32^, ^33^

Several emerging low‐cost and portable automated refraction and spectacle‐fitting technologies have been used in resource‐limited settings, including the SV One (Smart Vision Labs, smartvisionlabs.com), InstaRef (Remidio Innovative, remidio.com), QuickSee (PlenOptika, plenoptika.com), ClickCheck (EssilorLuxottica, essilorluxottica.com/en/newsroom/press‐releases/study‐confirms‐efficacy‐clickcheck/) and Plusoptix (Plusoptix GmbH, plusoptix.com/vision‐screener/).^34^ Reported mean differences relative to subjective refraction were −0.24 D for SV One,^35^ 0.14 D for InstaRef^36^ and 0.19 D for QuickSee.^37^ For the QuickSee instrument, a randomised crossover trial found that prescriptions from this low‐cost, portable autorefractor yielded visual acuity and patient acceptance levels comparable to those of subjective refraction, with no clear preference between the methods—supporting its use in resource‐limited settings.^16^ ClickCheck showed high agreement with subjective refraction for spherical power (ICC = 0.94) and moderate agreement for cylindrical power (ICC = 0.49).^38^ The SV One and Plusoptix showed a tendency towards more myopic estimates compared with subjective refraction.^39^, ^40^

In terms of self‐refraction, EyeNetra (EyeNetra Inc., eyenetra.com/) uses a smartphone to project visual targets and collect patients' subjective responses,^19^, ^20^ but it overestimates myopia by 0.27–0.53 D on average compared with subjective refraction (SR), with a SD of 1.40 D.^20^, ^41^ Kumar et al. noted that the spherical equivalent values measured by EyeNetra were within 0.25 D of the subjective refraction results in only 15% of the tested eyes,^42^ and the degree of myopia measured without pupil dilation was approximately 1.25 D higher than the actual value.^19^ The Easee eye (Easee B.V., easee.online/) test is an online self‐refraction tool. Although it can perform refraction through web pages and smartphone screens, there are significant differences between its results and traditional subjective refraction, and the measurement takes a relatively long time (an average of 22 ± 10 min).^43^, ^44^ Adjustable lenses can also be used for self‐examinations and can be locked after the examination to provide spectacle‐fitting prescriptions.^45^ However, subjects need to go through a challenging process to adjust the sphere, cylinder and axis for each eye.^17^, ^18^

Traditional symbolic pipelines translate images or wavefronts into refractive components via optical rules and analytic fits; they are transparent and theoretically grounded but behave approximately linearly in practice, so pushing precision typically requires premium sensors and tightly controlled conditions. By contrast, TRDS adopts a connectionist deep‐learning paradigm that learns a non‐linear mapping from image features to lens parameters and optimises an end‐to‐end objective tied to visual quality, thereby helping to relax the cost–accuracy frontier by extracting robust features from inexpensive signals, and compensating for some measurement imperfections. Concretely, TRDS employs a low‐cost CMOS detector, an infrared‐LED source and a simplified, no‐moving‐parts optical design (hardware cost < USD 150) with active sensing, enabling portable, open‐field operation without dark rooms or head hoods; the model was trained on 362,000 infrared eccentric photorefraction images to improve adaptability across pupil states, ocular surfaces and refractive types. The current data show strong agreement in low (*r* = 0.90) and moderate myopia (*r* = 0.88). No claim of superiority is made by downplaying other devices that already achieve clinically acceptable agreement under defined conditions; rather, TRDS is positioned as complementary—a methodological (algorithmic) advance that produces SR‐level performance using ultra‐low‐cost hardware while reducing dependence on premium optics and extensive operator training.

Compared with traditional infrared eccentric refractometers (such as the VS100), TRDS has advantages in the detection of astigmatic parameters. The intraclass correlation coefficients (ICC) of *J*_0_ and *J*_45_ were 0.71 and 0.75, respectively, which were significantly higher than the values of 0.32 and 0.40 obtained with the VS100, indicating that its ability to detect astigmatism is closer to the gold standard of subjective refraction. However, correlations of the *M* parameter between the TRDS and SR in the high myopia (*r* = 0.62) and low hyperopia groups (<+1 D) (*r* = 0.48) were slightly lower, which may be related to the relatively small sample size of the model training in these two groups. These subgroup differences provide directions for subsequent model optimisation, such as optical path calibration for high myopia or optimised compensation algorithms for the hyperopic group.

The reason for these improvements is that retraining the model with more targeted data allows it to capture better the intrinsic features and distributions of the new dataset, thereby enhancing its accuracy for specific populations. For example, in elderly subjects, reduced lens transparency leads to differences in the infrared retinal reflex compared with younger individuals. Because training an end‐to‐end deep learning model requires large amounts of data, a more efficient approach is to employ transfer learning: First, pretrain a general infrared‐eccentric refraction prediction model on the large‐scale dataset constructed here, and then fine‐tune it using refraction data from the target population. This strategy lowers the data‐scale requirements for the end‐to‐end model. Next‐generation hardware improvements may include integrating a dedicated neural‐network processing chip to localise the model on‐device, eliminating dependence on network or cloud computing. Since dedicated neural‐network chips offer significant performance and efficiency advantages over general‐purpose processors, the resulting device cost remains substantially below that of current commercial portable autorefractors.

A limitation of the present study is the small number of eyes with hyperopia >+2.50 D, reflecting the distribution of refractive errors in this 18–60‐year‐old outpatient cohort. Because high hyperopia is associated with a diminished infrared retinal reflex and smaller pupils, the current model—which was predominantly evaluated on myopic and low‐hyperopic data—may be less robust in this subgroup. Future studies will specifically recruit subjects with hyperopia >+2.50 D and apply transfer learning on hyperopia‐enriched datasets to fine‐tune the network. Moreover, multicentre trials in paediatric and geriatric populations are planned, where high hyperopia is more prevalent, to validate and optimise TRDS performance across the entire refractive error spectrum.

The clinical value of TRDS lies not only in technological innovation but also in its response to global eye health goals. The low‐cost and easy‐to‐operate characteristics of TRDS make it a powerful tool for improving eREC. It is particularly suitable for promotion in community health institutions, schools and other scenarios. By training non‐professionals to perform preliminary screening and generate prescriptions, it can relieve the shortage of professional resources. The highly consistent corrected visual acuity performance of TRDS and subjective refraction in this study lays a foundation for its large‐scale application in resource‐limited areas.

In conclusion, this study demonstrates the clinical value of a deep learning‐empowered portable refraction system in refractive error correction. It is expected to accelerate the transformation of refractive error correction from relying on professionals to an intelligent and inclusive model.

## AUTHOR CONTRIBUTIONS

**Huang Yan:** Data curation (lead); investigation (lead); writing – original draft (equal). **Zhen Yi:** Conceptualization (equal); formal analysis (equal); methodology (equal); resources (lead); supervision (lead); writing – original draft (equal); writing – review and editing (equal). **Sun Qilin:** Formal analysis (equal); investigation (equal); methodology (equal); software (lead); writing – original draft (equal). **Chang Hong:** Investigation (equal); methodology (equal); resources (equal); writing – review and editing (equal). **Huang Yan:** Software (equal); writing – original draft (equal); writing – review and editing (equal). **Tang Wei:** Conceptualization (equal); resources (equal); writing – original draft (equal); writing – review and editing (equal).

## FUNDING INFORMATION

This study was funded by the Beijing Municipal Science and Technology Project (Z201100005520042).

## CONFLICT OF INTEREST STATEMENT

All other authors declare no competing interests.

## REGISTRATION

The clinical trial was registered at clinicalTrials.org under the number ChiCTR2500100874.
